# Dynamic control of carbon‐ion radiation dose distribution amid respiratory motion challenges: A review of prediction research using geant4 and deep learning physics‐constrained models

**DOI:** 10.1002/acm2.70766

**Published:** 2026-10-20

**Authors:** Jianhua Ding, Peng Li, Chai Hong Yeong

**Affiliations:** ^1^ School of Medicine Faculty of Health and Medical Sciences Taylor's University Subang Jaya Malaysia; ^2^ School of Computing & Technology Asia Pacific University of Technology & Innovation Kuala Lumpur Malaysia

**Keywords:** carbon‐ion radiotherapy, computer‐assisted, deep Learning, Monte Carlo method, radiotherapy planning, respiratory motion

## Abstract

**Background:**

Carbon ion radiotherapy (CIRT) offers superior dose conformity and biological effectiveness; however, its precision is highly sensitive to respiratory motion, leading to dose uncertainty and interplay effects that compromise treatment accuracy.

**Methods:**

A comprehensive literature synthesis was conducted on studies employing motion‐resolved imaging techniques (e.g. SMEIR), dynamic dose prediction, 4D robust planning, and hybrid adaptive methods for CIRT. The study emphasised the use of Geant4 Monte Carlo simulations combined with deep learning models, including physics‐informed neural networks, to simulate and predict dose perturbations caused by respiratory motion. This review also examined clinical treatment planning systems, radiobiological modelling, and adaptive strategies to mitigate the effects of motion.

**Results:**

The integration of Geant4 with deep learning enabled high‐fidelity, computationally efficient dose prediction under respiratory motion, improving accuracy in dose localization and adaptation. Motion management techniques such as respiratory gating, rescanning, and 4D robust optimization demonstrated enhanced tumor control and normal tissue sparing. Hybrid models combining Monte Carlo data and AI inference show promise for near‐real‐time adaptive therapy. Despite these advances, challenges remain, including limited motion‐rich datasets, computational demands for real‐time recalculations, and the complexity of incorporating variable relative biological effectiveness (RBE) into adaptive planning.

**Conclusions:**

Combining Geant4‐based Monte Carlo simulations with deep learning frameworks shows emerging promise for controlling carbon ion dose distributions amid respiratory motion, though direct clinical validation for this combination remains limited. These hybrid models may enhance the precision and adaptability of CIRT, offering improved outcomes. Future efforts should focus on clinical validation, standardization, and overcoming computational and data limitations to facilitate clinical translation of motion‐adaptive CIRT. These approaches remain at Technology Readiness Levels (TRL 3–5) and require prospective clinical validation before routine implementation.

## 1 INTRODUCTION

In cancer radiotherapy, particle therapy—which includes techniques like proton and heavy ion beams—has emerged as a promising and increasingly adopted modality, supported by a growing body of clinical evidence, although robust phase III trial data remain limited and particle therapy currently treats only a small fraction of radiotherapy patients worldwide.^1^, ^2^ Unlike conventional photon‐based techniques, which distribute dose along the entire beam path according to an exponential attenuation profile, particle beams exploit the Bragg peak effect and can cause maximum energy deposition at a given depth with minimal total dose delivered, a considerable saving of normal tissues located out of the target.^1^, ^2^ Among particle therapies, carbon ion radiotherapy (CIRT) is distinguished from proton therapy by its greater linear energy transfer (LET) and relative biological effectiveness (RBE), making it specifically effective in the treatment of hypoxic, radioresistant or deeply seated tumours.^3^, ^4^, ^5^ Long‐term clinical data from facilities in Japan, Germany, and Italy suggest potential advantages of carbon ions in treating difficult cancers such as sacral chordomas, head‐and‐neck tumours, and pancreatic cancers, although direct randomized comparisons against photon therapy remain very limited.^2^

The therapeutic advantage of carbon ions, however, is very dependent upon accurate dose deposition. Small differences between the planned and actual dose of deliveries can result in considerable clinical impact because of large dose rates at the Bragg peak and increased biological responses at distances close to the distal edge, even in cases when there is only a small beam range uncertainty.^6^ Accuracy of dose distributions, therefore, is a fundamental precondition in CIRT, which requires effective treatment planning systems and quality assurance procedures.^7^ Respiratory motion presents a major challenge in thoracic and abdominal radiotherapy because it introduces time‐varying anatomical displacements in multiple spatial directions simultaneously. Diaphragm and chest‐wall excursion is commonly reported in the range of 5–25 mm, but tumor displacement itself is generally smaller and highly site‐specific: most lung tumors move less than 10 mm, whereas liver tumors—particularly those near the diaphragm—can exceed 20 mm in the superior‐inferior direction; breathing frequency (∼12–20 per min) is randomly variable, especially in stress, when sedated, or with a disease.^8^ While the tumor itself does not change in volume during respiration, the surrounding anatomy deforms around it, shifting its position and the density of tissue traversed by the beam, which creates a dynamic requirement for compensation techniques. As an example, the superior‐inferior motion of liver tumor can be greater than lateral movement and could be greater than 20 mm, whereas direction dependent deformation of lung tumor could be present during the breathing process.^9^ During respiration, the position of the tumour and the surrounding anatomical structures may undergo deformation, particularly in thoracic and abdominal regions. Modern tumour tracking systems and surface‐guided radiotherapy (SGRT) are capable of detecting abnormal respiratory patterns in real time, allowing for the interruption of beam delivery through respiratory gating. While these methods enhance geometric accuracy, they typically result in an increase in overall treatment time. Abnormal breathing patterns like sighing and coughing introduce uncertainties to the dose tracking—that is, the time‐integrated accumulation of dose across respiratory phases relative to a reference anatomy—of scanning ion beams.

The interplay effect is considered one of the most significant dose distribution issues in CIRT, a phenomenon produced by the interaction between dynamic delivery of the beam (e.g. spot scanning) and organ motion. Since the target shifts during the respiration cycle or cardiac cycles, the accurately targeted carbon ion spots will not fall on the desired point, causing under‐ or over‐dosage in the tumor volume.^10^ The research highlights the prominent nature of this interaction in particle therapy as it is especially sensitive to spatially derived changes, which makes it a major aspect in planning quality.^11^ Besides the interplay, range uncertainties due to motion are still a cause of serious concern. Because carbon ions deposit the bulk of their energy sharply at the Bragg peak with minimal distal dose, any anatomical changes during breathing or digestive movements can shift the Bragg peak out of the tumour, possibly irradiating normal tissue.^9^ In CIRT specifically, positional changes and set‐up errors in deep‐seated or thoracic tumours have consequences beyond simple geometric miss: because the beam range is highly sensitive to the density of tissue traversed, even small anatomical shifts can displace the high‐dose region into or out of the target volume, underscoring the need for careful pre‐treatment analysis.^12^

Breathing and other physiological motions lead to complex patterns of dose deviations, which are not easily predicted by using simple, static 3D planning, and further highlight the necessity of incorporating temporal variations to dosimetry. These challenges have necessitated adaptive and robust planning methodologies. Multi‐phase 4D delivery approaches synchronize the beam to specific respiratory phases to minimize interplay,^13^ whereas 4D‐robust optimisation techniques instead feature explicit representation of varied uncertainties (i.e. patient anatomy shift, range error) directly within the plan objective, without requiring synchronization to a specific motion phase, in an attempt to limit interplay and motion effects.^14^, ^15^ A comprehensive overview of motion mitigation strategies in particle therapy, spanning gating, rescanning, and robust and 4D optimization, is provided by Galeone et al. (2025). High‐end technologies (e.g., multi‐phase 4D radiotherapy, respiratory gating) are also useful in this respect because they synchronize beam delivery with patient breathing, minimizing effects of interplay and motion uncertainties.^16^ There is growing interest in in‐vivo range verification strategies—including PET‐based detection of positron‐emitting fragments, prompt‐gamma imaging, and radioactive ion beams to minimise the safety margins traditionally required for range uncertainties, thus improving conformity.^7^, ^17^

Moreover, there is a growing interest in leveraging advanced computational modeling and artificial intelligence (AI). The use of Monte Carlo (MC) simulations with platforms such as Geant4 have become essential in the realistic simulation of dose distributions in complex clinical conditions, such as the presence of respiratory motion. These kinds of simulations permit high‐fidelity dose calculations which consider heterogeneities of tissues as well as the stochastic interactions of the particles. Several general‐purpose Monte Carlo toolkits—including FLUKA, TOPAS, GATE, and MCNPX—are also used in particle therapy dosimetry. Geant4 was selected as the focus of this review because it uniquely combines macroscopic dose simulation with the Geant4‐DNA extension for nanodosimetric and track‐structure modelling, and because the Geant4 collaboration maintains the G4‐Med validation platform, which benchmarks the toolkit's physics models specifically for medical applications, including carbon ion therapy. This combination of clinical‐scale dose calculation and biologically resolved modeling within a single, actively validated framework distinguishes Geant4 from other general‐purpose codes for the purposes of this review. In parallel, deep learning methods are being investigated to quickly propagate dose distributions from anatomical and motion information, with recent work focusing on physics‐informed neural networks that naturally produce physically valid results by applying known dosimetric constraints. Notably, hybrid methods that combine Monte Carlo data and deep learning, including CAM‐CHD U‐Net heavy‐ion plans or CNN‐based dose denoisers for proton/cyberknife systems, seek to provide the superior accuracy of physics‐based models, with the speed of AI inference, to permit real‐time and adaptive radiotherapy workflows.^18^, ^19^ Dedicated architectures for online adaptive particle therapy, such as DOTA and C‐DOTA, have further been proposed specifically for dose prediction under motion in carbon ion beams. It is against this background that the present review aims to critically synthesize the existing literature devoted to the development of methods of dynamical control of carbon ion dose distributions on the back of respiratory motion, with a specific focus on the use of prediction models that combine the capabilities of Geant4‐based Monte Carlo simulations and deep learning or physics‐constrained modeling frameworks. This review provides a critical evaluation of the methodologies, reporting of the most relevant findings, and the identification of the challenges that remain open, thereby becoming a useful source to clinicians, physicists and researchers at the intersection of particle therapy, computational modeling, and AI‐based adaptive therapy approaches. The challenges associated with FLASH radiotherapy are anticipated to intensify, given the ultra‐high dose‐rate delivery characteristic of this modality. This delivery method may exhibit heightened sensitivity to respiratory motion and interplay effects, thereby underscoring the necessity for precise motion prediction and adaptive dose control.

## 2 METHODS

This structured narrative review was informed by a systematic literature search across PubMed, Scopus, ScienceDirect, Google Scholar, and Web of Science, focusing on studies published from January 2010 to March 2026. The search strategy involved combining terms like “carbon ion radiotherapy” or “heavy ion therapy” with “Geant4” or “Monte Carlo simulation”, alongside “respiratory motion”, “4D”, or “interplay effect”, and “deep learning”, “machine learning”, or “artificial intelligence”. Additional keywords included “SMEIR”, “relative biological effectiveness”, and “treatment planning system”. To uncover further pertinent studies, reference lists from qualifying articles and recent reviews were manually examined.

Studies were selected if they presented original data on computational, dosimetric, imaging, treatment‐planning, or clinical aspects related to Geant4‐based or similar Monte Carlo simulations, respiratory motion management, 4D dose calculation, adaptive radiotherapy, or AI‐assisted dose prediction in particle therapy, particularly focusing on carbon ion radiotherapy. Review articles were mainly used to provide methodological context and to pinpoint primary studies, but they did not serve as the main evidence for specific conclusions. Exclusions were made for non‐English publications, conference abstracts without full‐text articles, and studies that lacked adequate methodological detail.

Due to the diverse nature of the available evidence, which included computational, phantom, imaging, treatment‐planning, and clinical studies, a formal systematic review, PRISMA‐guided quantitative synthesis, or risk‐of‐bias assessment was not conducted. Instead, the chosen literature was critically analyzed to assess recent advancements in Geant4‐based Monte Carlo simulation, motion‐resolved imaging, respiratory motion management, radiobiological modeling (LEM and MKM), deep learning‐based dose prediction, and hybrid adaptive strategies for carbon ion radiotherapy. The review was conducted as a structured narrative review and therefore did not follow PRISMA guidelines because of the heterogeneous computational, phantom, dosimetric, and clinical evidence included.

A total of 312 records were identified through searching (PubMed, Scopus, ScienceDirect, Web of Science, and Google Scholar). After duplicate removal (n = 48), 264 publications remained for title and abstract screening. Following screening, 147 articles unrelated to carbon‐ion radiotherapy, respiratory motion management, Monte Carlo simulation, or artificial intelligence‐based dose prediction were excluded. The remaining 117 full‐text articles were assessed for eligibility; 82 publications were included in the narrative synthesis after excluding review articles, conference abstracts without details, and studies outside the scope. Quantitative meta‐analysis was inappropriate because of heterogeneity.

## 3 BACKGROUND: CARBON ION RADIOTHERAPY—PHYSICS, RADIOBIOLOGY, AND MOTION CHALLENGES

The physics and radiobiology of carbon‐ion therapy are well established in the literature and are not, in themselves, novel findings of this review. They are summarised here only to the extent needed to interpret the motion‐management, Geant4‐based simulation, and deep learning literature that follows, and the treatment below is deliberately condensed relative to standard reference texts on the subject. Readers already familiar with Bragg‐peak physics, LET, RBE, LEM, and MKM may proceed directly to Section 3.2, which addresses the motion‐specific background most directly relevant to this review, or to Section 4, where the critical synthesis of the reviewed literature is presented.

### 3.1 Principles of carbon ion radiotherapy

#### 3.1.1 Physics of carbon ion radiotherapy

Carbon ion radiotherapy exploits the Bragg peak effect, depositing the bulk of beam energy at a precise depth and sparing surrounding tissue relative to the exponential attenuation profile of conventional photons.^20^ Figure 1 demonstrates the effect of respiratory motion on this precise dose localization, which is the central motion‐related vulnerability examined throughout this review. Modern scanning‐beam technologies overlay multiple Bragg peaks to create a spread‐out Bragg peak (SOBP) with consistent dose coverage across extended tumour volumes^21^; as discussed in Section 5, the LET—and therefore the biological effectiveness—varies considerably across this SOBP, which has direct implications for motion‐affected planning.

**FIGURE 1:**
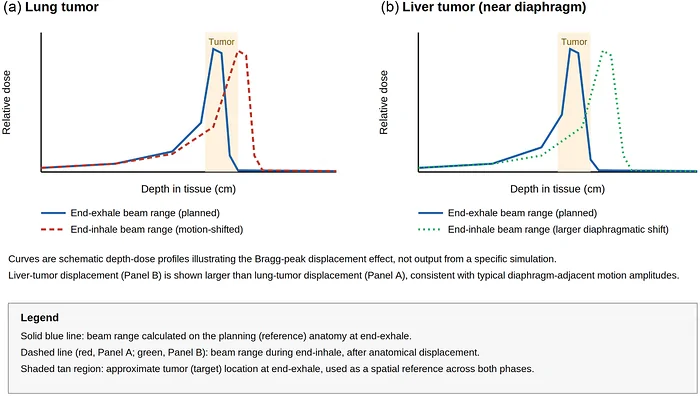
Schematic illustration of how respiratory motion impacts Bragg peak alignment during carbon ion radiotherapy in lung tumors.

Carbon ions attain LET values approaching ∼100 keV/µm only at the distal edge of a clinically realistic SOBP, with considerably lower LET—and correspondingly lower biological effectiveness—in the proximal and mid‐SOBP regions; this spatial LET variation, and the densely ionised, less repairable DNA damage it produces, underlies the biophysical planning approaches discussed below.^22^, ^23^, ^24^ Nuclear fragmentation also produces a low‐dose fragmentation tail distal to the target that must be accounted for in dose calculations; this is a dosimetric liability rather than the planned therapeutic benefit some earlier literature has speculated.^25^

#### 3.1.2 Radiobiology of carbon ions

Carbon ions' high LET generates clustered double‐strand breaks largely independent of oxygenation status or cell cycle phase, making them effective against hypoxic or radioresistant tumours where photon therapy often underperforms.^26^, ^27^ Carbon‐ion RBE varies between approximately 2–5 depending on target tissue, biological endpoint, and LET,^28^ and—unlike proton therapy's fixed clinical RBE of 1.1—varies spatially within the field, peaking near the distal Bragg‐peak edge. Modern planning systems accommodate this spatial variation using the Microdosimetric Kinetic Model (MKM) and Local Effect Model (LEM),^21^ discussed further below, which several comparative studies link to improved tumour control in radioresistant disease.^23^, ^29^ A comparison of key physical and biological parameters across photon, proton, and carbon‐ion therapy is provided in Table 1.

**TABLE 1:** Comparison of physical and biological parameters of different radiation modalities.

Parameter	Photons	Protons	Carbon Ions
LET (entrance, keV/µm)	∼0.3	∼0.5	∼10–20
LET (Bragg peak / distal SOBP edge, keV/µm)	N/A (no Bragg peak)	∼8–10	∼70–100
Relative Biological Effectiveness (RBE)	1.0 (reference)	∼1.1 (fixed, clinical convention)	Spatially variable, ∼2–5 (LEM/MKM‐modeled)
Dose deposition profile	Exponential decay with depth	Sharp Bragg peak, sharp distal fall‐off	Sharp Bragg peak, sharp distal fall‐off, with a low‐dose fragmentation tail
Nuclear fragmentation tail	Not applicable	Minimal / negligible	Present (secondary fragments distal to target)
DNA damage character	Sparse ionization, predominantly single‐strand breaks, more repairable	Sparse‐to‐moderate ionization density	Dense ionization, clustered double‐strand breaks, less repairable

#### 3.1.3 Clinical treatment planning

In clinical practice, CIRT requires biologically informed treatment planning, which is significantly more complex than that for photon or proton therapy. Treatment planning systems (TPS) must integrate both physical dose calculations and biological parameters such as LET and RBE. TRiP98, developed at GSI Darmstadt, was used during the original German pilot clinical project but is no longer licensed for routine clinical use; it was succeeded by Siemens Syngo PT, which remains operational at some centers. RayStation (RaySearch Laboratories), which supports both the LEM and MKM biophysical models, is now the dominant treatment planning system used across the majority of carbon ion facilities in Europe and Japan. For instance, in sacral chordoma patients, LEM‐based RBE‐weighted dose combined with dose‐averaged LET was shown to significantly affect local control and normal tissue sparing.^30^ Critically, carbon‐ion RBE is not a function of LET alone: it also depends on dose per fraction, tissue radiosensitivity (α/β ratio), and biological endpoint, which is why LEM and MKM are needed to translate the spatially varying physical dose and LET into a clinically meaningful RBE‐weighted dose rather than simply maximizing physical dose to the target.^23^ An additional source of uncertainty arises from the α/β ratio used in radiobiological models. Published α/β values may differ by approximately 30% depending on tumour type, experimental conditions, and model assumptions, which can substantially influence predicted RBE‐weighted dose distributions and treatment optimisation.

Góra et al.^29^ proved that treatment conclusions, such as dose per fraction and CTV coverage, vary significantly depending on whether LEM or MKM is used, highlighting the pragmatic implications of model choice. Mein et al.^31^ validated RBE‐weighted models across three levels, revealing that outdated mid‐LET assumptions can overestimate toxicity risk, necessitating TPS modernization.

The next frontier is multi‐objective optimization. Jointly optimizing physical and biological dose, including nanometric ionization‐cluster distributions, was explored in the 2000s; Burigo et al.^32^ provided a computational protocol for simultaneous optimization of RBE‐weighted dose and nanometric distributions. At very high LET (above ∼200 keV/µm), the overkill effect must be accounted for in biophysical models, particularly near the distal Bragg peak. Precise model translation aids in treating difficult tumors like adenoid cystic carcinoma (ACC). The debate between LEM and MKM versions involves dosimetric and clinical comparisons. Molinelli et al.^33^ found no clinically significant differences in ACC outcomes between LEM and MKM, underscoring the difficulty of isolating model‐specific effects. Range uncertainty remains a primary challenge, especially in motion‐affected sites, prompting increased use of adaptive re‐planning and motion‐managed delivery. There is growing interest in hybrid planning integrating photon and carbon ion plans. Bennan^34^ addressed optimal LET and RBE for complex volumes, using carbon ions to increase biological effect in resistant areas and photons to manage geometric uncertainty. Weyrather and Kraft^35^ established the basis for LEM‐based planning by comparing in vitro RBE data with predictive models, a contribution still applied in TPS calibration today.

### 3.2 Respiratory motion in carbon ion radiotherapy

Respiratory motion is complex, defined by amplitude, frequency, and irregularity. These parameters impact radiation therapy, diagnostics, and biomechanics. Amplitude, the displacement of thoracic or abdominal structures, is patient‐ and region‐specific; most lung tumors move under 5–10 mm^36^. Precise quantification is vital in radiation therapy to avoid geographic miss^36^. In stroke patients, irregular amplitude disrupts breathing coordination, affecting vocalization and cough reflexes.^37^

Healthy adults breathe 12–20 times per minute, though rates shift with exercise, stress, or pathology. Frequency influences chest wall and diaphragmatic movement, impacting mechanical ventilation. Altered patterns often indicate underlying pathology or brain stem dysfunction^38^, ^39^ demonstrated that respiration's oscillatory nature, where impedance and phase change with frequency, is essential for modeling lung diseases like asthma and COPD. Abnormal breathing may stem from neurological illness, anxiety, or cancer. Cheong et al^40^ proposed the respiration regularity index (RRI) to measure amplitude and period variability, improving radiotherapy gating. In panic disorder patients,^41^ linked respiratory chaos to mental health and autonomic dysregulation. Modeling confirms respiratory motion is not purely sinusoidal, involving baseline drift, phase shifts, and frequency modulations that challenge clinical gating and tracking systems.^42^ Compared with breath‐hold or respiratory gating techniques, adaptive motion modelling allows treatment under free breathing and may improve patient comfort while reducing dependency on patient compliance. However, breath‐hold and gating generally increase treatment delivery time and workflow complexity.

Carbon ions have a steep Bragg peak and great sensitivity to density and path‐length variations, as well as higher mass. This increases their vulnerability to misalignment and underdose or overdose, unlike photons, which have diffusive profiles of dose deposition, or even protons, which, despite their lower LET are typically planned using a fixed clinical RBE of 1.1 regardless of position—a simplification that eases planning but is increasingly questioned, in contrast to CIRT, where the higher and spatially variable LET requires explicit, position‐dependent RBE modeling.^43^ In actively scanned beam delivery, interplay between tumor motion and pencil beam scanning (PBS) can cause dose distortions—called the interplay effect—where dose lines miss the tumor during respiration, reducing target conformity. Richter et al.^44^ demonstrated that motion amplitudes as small as 6 mm in liver tumors could significantly degrade dose homogeneity, but increased pencil beam overlap mitigated this effect. Chi et al.^45^ compared intensity‐modulated carbon ion therapy (IMCIT) to IMPT and VMAT under simulated lung tumor motion; however, respiratory gating was applied to restrict motion to ≤5 mm, and interplay effects—to which carbon ions are the most sensitive of the three modalities studied—were not modeled. Their results should therefore be interpreted cautiously and do not support a general claim of carbon‐ion robustness at amplitudes up to 20 mm.^45^

The sensitivity of the dose distribution to respiratory motion varies widely across carbon ion, proton, and photon therapies, with carbon ion therapy being the most sensitive owing to its steep dose gradient and biological effectiveness (Table 2)

**TABLE 2:** The essence of the effect of respiratory motion on the movements of carbon ions, protons, and photons regarding essential parameters and clinical implications.

Consideration under respiratory motion	Photons	Protons	Carbon Ions
Lateral dose falloff	Gradual, geometry‐ and energy‐dependent	Sharper than photons at depth in many configurations	Sharper than photons at depth in many configurations
Consequence of a misplaced Bragg peak / range error	Not applicable (no Bragg peak); dose displacement is primarily lateral	Distal dose deposited beyond or short of target; clinically significant but generally lower biological consequence than carbon	Distal dose deposited beyond or short of target; clinically significant, with potentially greater biological consequence due to higher RBE at the point of error
Sensitivity to interplay effect (motion × scanned‐beam delivery)	Lower (typically delivered with broad fields, not spot‐scanned)	Moderate	Highest among the three modalities, due to sharp dose gradients and high LET sensitivity to small positional shifts
Effect of motion‐induced dose smearing	Present; a function of tissue motion relative to the static dose distribution, compensated by techniques such as IMRT/VMAT motion management	Present; requires motion mitigation (gating, rescanning, robust optimization)	Present; requires motion mitigation, with added sensitivity due to range/density dependence of the Bragg peak

CIRT is clinically beneficial in lung and liver cancers (Table 3), particularly in the treatment of hypoxic or radioresistant lesions, where a high LET is desirable. Nevertheless, the respiratory‐induced displacements were greatest at these locations. The inferior‐superior movement of liver tumors, for example, exceeding 30 mm, makes it difficult to accurately target the beam during scanning of the beam.^44^ In lung tumors, single‐fraction stereotactic body radiation therapy (SBRT) as well as fractionated with carbon ions have been studied. Anderle et al.^46^ determined that carbon ions and photons had better normal tissue sparing even in the setting of simulated respiratory motion by utilizing carbon. This result was attributed more to a less integral dose. Motion effects are progressively addressed using adaptive approaches, including respiratory gating, beam rescanning, and 4D robust optimization. These methods either time the delivery of the beams to coincide with particular phases of respiration or stochastically average motion‐induced errors across an entire series of beam passes.^9^, ^47^ Clinically, respiratory gating is most commonly performed during the end‐expiration phase because respiratory motion is generally more reproducible and stable than during inspiration, thereby improving tumour localisation accuracy.

**TABLE 3:** Carbon Ion Radiotherapy in Lung vs. Liver Tumors under Respiratory Motion.

Tumor site	Typical motion amplitude	Key clinical consideration	Boundary/overlap cases
Lung (peripheral)	Generally < 5–10 mm	Motion mitigation often manageable with rescanning or gating	Apical tumors near the chest wall behave differently from basal tumours
Lung (mediastinal/central)	Often < 5 mm	Motion may be small, but proximity to critical structures (heart, oesophagus, major vessels) is the dominant constraint rather than motion itself	Overlaps with cardiac motion considerations; not well captured by a simple "lung" category
Liver (general)	Can exceed 20 mm, particularly superior‐inferior	Requires robust motion management; ITV or gating strategies are common	—
Liver dome (adjacent to diaphragm)	Often, the largest amplitudes are observed clinically, exceeding 30 mm in some patients	The greatest risk of Bragg peak displacement due to coupling with diaphragmatic excursion	A liver tumour extending toward the lung base blurs the liver/lung distinction entirely and may require combined motion modeling

## 4 RESEARCH TRENDS

Although numerous studies have independently investigated Geant4‐based Monte Carlo simulation, respiratory motion management, and deep learning dose prediction, relatively few studies explicitly integrate all three components into a unified carbon‐ion adaptive radiotherapy framework. Of the literature identified through our search strategy, fewer than ten studies explicitly combine Geant4‐generated or Geant4‐comparable Monte Carlo dose data with a deep learning or physics‐constrained prediction model in the specific context of respiratory motion in carbon‐ion therapy (e.g.,^18^, ^48^; the DOTA and C‐DOTA architectures of Wahl et al. 2026, 2026b); a larger but still modest set of studies addresses two of the three components—for instance Geant4‐based 4D dose calculation without deep learning,^48^ or motion‐resolved imaging coupled with non‐Geant4 dose engines^49^, ^50^—and these are included for context rather than as direct evidence for the integrated approach. This small and heterogeneous evidence base is itself informative: it indicates that the explicit combination of Geant4, deep learning, and respiratory‐motion management for carbon‐ion therapy is an emerging rather than an established research direction, which motivates a narrative synthesis over a fully quantitative review at this stage, while also underscoring the need for further dedicated studies at this intersection.

Respiratory motion introduces significant challenges in CIRT due to the precise dose localization associated with the Bragg peak of heavy ions. Therefore, multiple studies have explored advanced dose calculation tools, such as Geant4‐based Monte Carlo simulations, deep learning (DL) models, and the Simultaneous Motion Estimation and Image Reconstruction (SMEIR) framework, to accurately model or mitigate the effects of motion interplay during treatment (Table 4).

**TABLE 4:** Summary of Geant4‐based Monte Carlo, dose engines, motion‐resolved imaging, and deep learning approaches for dose calculation and prediction in carbon‐ion radiotherapy under respiratory motion.

Study	Dose Engine / Method	Motion‐Handling Approach	Key Finding
Dowdell et al. ^[^ ^48^ ^]^	Geant4 (4D‐MC)	4D‐CT‐based simulation of breathing motion	Substantial dose smearing in the superior‐inferior direction without motion mitigation
Shrestha et al. ^[^ ^50^ ^]^	goCMC (not Geant4)	SMEIR‐based 4D‐CBCT reconstruction	Improved dose‐calculation accuracy from motion‐resolved anatomy, plausibility‐validated on SMEIR‐reconstructed images
He et al. ^[^ ^18^ ^]^	Deep learning trained on Monte Carlo data	4D‐CT‐derived motion data for heavy‐ion adaptive RT	High‐fidelity, near‐real‐time dose prediction relative to full Monte Carlo at substantially reduced computational cost
Reidel et al. ^[^ ^55^ ^]^	Geant4	4D‐online density‐gradient monitoring	Demonstrated feasibility of online motion monitoring for CIRT dose verification
Marhou et al. ^[^ ^57^ ^]^	Geant4	1.5T MRI‐guided CIRT simulation	Magnetic field coupling with respiratory motion modulates Bragg peak position via lateral shifts

The role of Geant4‐based Monte Carlo simulation in this context, including its 4D simulation capabilities and known limitations, is discussed in full in Section 5, which consolidates material previously split across two sections in response to reviewer feedback.

### 4.1 SMEIR‐based dose reconstruction

SMEIR (Simultaneous Motion Estimation and Image Reconstruction) is an advanced imaging technique that reconstructs 4D images from cone‐beam CT data while simultaneously estimating anatomical motion. It is important to note that SMEIR is an image‐domain reconstruction algorithm, not a dose calculation engine: it provides the anatomical model on which a separate dose engine subsequently calculates dose. SMEIR can also be used in CIRT to provide clinicians with improved models of patient‐specific respiratory motion and to adjust dose calculations to the patient's respiratory motion, thereby providing a higher level of accuracy. In a study by Shrestha et al.^50^, the SMEIR for CIRT of lung cancer was assessed, and Shrestha et al.^50^ demonstrated that the use of SMEIR‐generated 4D cone‐beam CT significantly enhanced motion‐resolved anatomical reconstruction in carbon‐ion radiotherapy, facilitating more precise dose accumulation throughout the respiratory cycle. Subsequent validation by Annunziata et al.^49^ confirmed improved target localization and a reduction in organ‐at‐risk dose compared to conventional motion‐compensated reconstruction strategies. Nevertheless, the majority of published studies emphasize qualitative or workflow‐based improvements rather than standardized quantitative metrics, such as gamma pass rate, dose‐volume histogram (DVH) indices, target coverage (D95%), conformity index (CI), or computational efficiency. As a result, direct quantitative comparisons between SMEIR and Geant4‐based Monte Carlo dose calculations remain limited. Given that SMEIR is fundamentally an image‐reconstruction algorithm rather than a particle‐transport dose engine, its primary contribution lies in generating accurate motion‐resolved anatomy, upon which Geant4 or other Monte Carlo platforms can subsequently perform dose calculations. Therefore, current evidence supports SMEIR as a complementary imaging framework that enhances anatomical fidelity for adaptive carbon‐ion therapy. However, further studies reporting standardized quantitative dosimetric endpoints are necessary to fully establish its clinical benefits. Dose calculation in that study was performed using the goCMC Monte Carlo engine rather than Geant4^51^; SMEIR and Geant4 are conceptually distinct tools—an image‐reconstruction algorithm and a particle‐transport dose engine, respectively—and direct agreement between the two is therefore not the relevant comparison. Rather, the two are complementary: SMEIR (or an analogous motion‐resolved reconstruction method) supplies the time‐resolved anatomy, while a Monte Carlo engine such as Geant4 performs the dose calculation on that anatomy. Motion‐resolved approaches such as SMEIR are most valuable when the planning goal is to accurately accumulate the biologically weighted dose across respiratory phases, or where interplay cannot be adequately averaged out by rescanning; a properly constructed internal target volume (ITV) with density override on a static CT, combined with beam rescanning, can already achieve adequate dose coverage in many routine scenarios without requiring full 4D imaging or reconstruction. A summary of the reported performance outcomes, computational advantages, and current limitations of recent deep learning and motion‐adaptive approaches is presented in Table 5.

**TABLE 5:** Reported performance outcomes for recent studies on deep learning and motion adaptation in carbon‐ion radiotherapy.

Study	AI / Computational Method	Quantitative findings	Limitation
He et al. ^[^ ^18^ ^]^	Deep learning Monte Carlo surrogate	Near‐real‐time dose prediction with high agreement to Monte Carlo reference doses; substantial reduction in computation time	Limited patient cohort; retrospective validation
Shrestha et al. ^[^ ^50^ ^]^	SMEIR + 4D CBCT	Improved motion estimation and target localization; enhanced dose accumulation	Limited quantitative dosimetric comparison with Monte Carlo
Annunziata et al. ^[^ ^49^ ^]^	Virtual 4DCT	Improved organ‐at‐risk sparing and target alignment	Phantom and limited clinical validation
Dowdell et al. ^[^ ^48^ ^]^	4D Geant4 Monte Carlo	Demonstrated dose degradation under respiratory motion; highlighted the importance of motion compensation	Simulation study only
He & Mori ^[^ ^12^ ^]^	4D perturbation analysis	Quantified respiratory‐induced dose perturbations supporting adaptive planning	No deep learning integration
Galeone et al. ^[^ ^66^ ^]^	Real‐time delivered dose assessment	Demonstrated feasibility of real‐time delivered dose evaluation for moving targets	Clinical implementation remains limited

### 4.2 Deep learning and respiratory modeling

Deep Learning can be defined as a machine learning model that uses artificial neural networks to learn complicated patterns in a large dataset. In radiation oncology, DL has the ability to simulate non‐linear associations among anatomical movement and the amount of dose distribution, frequently in real‐time. When used together with physical modeling (e.g. physics‐informed neural networks or PINNs), DL can be used to generate clinically meaningful and physically plausible outputs. It should be clarified that Meschini et al.^52^ used an optical surface‐monitoring‐derived surrogate respiratory signal to guide gated CIRT, not a deep learning model; genuinely DL‐based approaches for CIRT dose prediction include He et al.^18^, who demonstrated deep learning–based Monte Carlo dose prediction for heavy‐ion online adaptive radiotherapy with high fidelity relative to full Monte Carlo calculations at substantially reduced computational cost. Moreover, DL frameworks are being applied to designing predictive analytics, which simulate operationally expensive models, such as Monte Carlo calculations, potentially permitting real‐time treatment adjustment without sacrificing precision. He et al. achieved a high level of agreement with Monte Carlo reference doses, significantly reducing computation time from several minutes to nearly real‐time inference. However, the validation was conducted on limited datasets, and prospective clinical validation has yet to be performed. Recent advancements in deep learning methodologies have significantly decreased Monte Carlo computation times while preserving high dosimetric accuracy. This finding suggests its potential application in online adaptive radiotherapy. However, the model's development relied on relatively limited training datasets and retrospective validation, with external multi‐institutional validation still lacking. Similarly, physics‐informed neural networks and U‐Net‐based architectures have shown enhanced robustness by integrating physical dose constraints during training, thereby minimizing prediction artefacts compared to purely data‐driven models. Despite these promising results, most studies are still limited to simulation environments, and prospective clinical implementation has not yet been realized. Present limitations encompass the scarcity of motion‐rich datasets, the uncertainty in generalizing findings across different institutions, the absence of standardized benchmarking datasets, the lack of prospective clinical validation, and the ambiguity surrounding regulatory implementation within adaptive carbon‐ion radiotherapy workflows.

While these studies consistently show enhanced computational efficiency and promising dosimetric accuracy, the overall readiness of the technology remains constrained. The majority of published research comprises proof‐of‐concept studies utilizing retrospective datasets or computational phantoms. To incorporate Geant4–deep learning frameworks into routine adaptive carbon‐ion radiotherapy, standardized benchmarking, prospective multicenter validation, workflow integration, and regulatory approval are necessary.

### 4.3 Comparative and hybrid studies

A comparative or hybrid model that integrates multiple modelling tools is frequently required to capture the interaction between motion and treatment delivery in CIRT.^53^ Such may comprise Geant4 simulations, SMEIR image‐based correction, and DL estimation devices. In a similar example, Anderle et al.^46^ conducted an in silico study to compare photon therapy and carbon ion therapy using simulated breathing motion with 4DCT. They proved that CIRT, although having a better target, is more prone to respiratory motion dose distortion owing to its sharp Bragg peak.

## 5 GEANT4 TOOLKIT OVERVIEW & RELEVANCE FOR CIRT

Accurate prediction of dose perturbations caused by respiratory motion is essential in particle therapy, especially in CIRT, which is highly sensitive to anatomical changes and intra‐fractional motion. Monte Carlo simulations, particularly those built using Geant4, offer detailed modeling capabilities to assess these perturbations with high fidelity. Geant4 is particularly well suited to simulating the interaction of high‐LET particles with tissues, which is an important aspect of CIRT due to its large physics libraries and modular capabilities. It is also complemented by its Geant4‐DNA extension to support nanodosimetric modeling and modeling of biological effects, an important consideration in analyzing cellular effects due to radiation.^54^ Geant4‐DNA specifically extends the standard toolkit to simulate particle track structure at the nanometric scale, enabling stochastic modeling of clustered DNA damage and supporting microdosimetric implementations of the MKM that underlie several clinical RBE models. Relevant physics developments for CIRT include the Liege Intranuclear Cascade model coupled to quantum molecular dynamics (LIQMD) and explicit treatment of multi‐ionization processes, both of which affect the nuclear interaction cross‐sections governing secondary‐particle production and, in turn, the predicted fragmentation tail. Because Geant4 simulation results are strongly dependent on the specific physics list and toolkit version used, the Geant4 collaboration maintains G4‐Med, a dedicated benchmarking platform that validates Geant4 physics models against experimental data for medical applications including CIRT; reviewers and users of Geant4‐based CIRT studies should consider which version and physics list were validated against G4‐Med before drawing comparative conclusions across studies (cite Geant4 collaboration documentation; e.g.).

Geant4 (Geometry and Tracking), developed by CERN, models microscopic physical interactions such as electromagnetic interactions, hadronic interactions, and nuclear processes, a level of granularity essential for estimating the Bragg peak and the complex dose distributions that accompany high‐LET particles in CIRT. Dowdell et al.^48^ developed a 4D Monte Carlo simulation using Geant4 to evaluate the interaction between breathing and particle beams, finding that dose smearing effects were considerably high in the absence of proper motion control, particularly in the superior‐inferior direction. This single early study, however, is illustrative of a broader gap: relatively few Geant4‐based 4D simulation studies for CIRT have been published, and direct quantitative comparisons between 4D Monte Carlo (4D‐MC) and 4D pencil‐beam algorithms (4D‐PBA) remain scarce. This is a meaningful omission, because Monte Carlo carries a particular computational advantage in 4D scenarios: 4D‐PBA requires separate analytical dose calculations for each respiratory phase or scenario, with computational effort scaling roughly linearly with the number of phases, whereas 4D‐MC can sample particle histories across the full motion envelope within a more unified simulation framework, without a proportional per‐phase increase in cost. Future Geant4‐based CIRT studies should explicitly benchmark 4D‐MC against 4D‐PBA in heterogeneous, motion‐affected anatomy to substantiate this advantage.

Geant4‐based simulations have proven effective in dynamic environments with respiratory motion.^55^ As an example, Andersson^56^ was able to leverage machine learning together with Monte Carlo modeling to enhance in vivo transmission dosimetry in dynamic therapies. With this hybrid technique, it was possible to model pixel‐specific dose‐response functions under realistic motion conditions. The need for such modelling is evident when one considers the implications of motion‐induced misalignment of the Bragg peak: the tumour is severely underdosed, while healthy tissues are overdosed. A study by Chatzisavvas & Priniotakis emphasizes the maturity of Monte Carlo toolkits as general‐purpose dose calculation tools in radiation dosimetry, involving dynamic anatomical settings, such as Geant4 and MCNPX. They noted that further development of simulation‐based verification systems is essential for motion‐influenced treatments.

In addition to respiratory motion, other perturbative factors have been studied in recent times, for example, an external magnetic field, which can potentially couple with respiratory motion in MRI‐guided CIRT. For example, Marhou et al.^57^ described a dose shift under the influence of a 1.5 T magnetic field during carbon therapy. Their simulations using Geant4 told of both lateral shifts and modulations of the Bragg peak with field orientation and respiratory phase, adding to the understanding of how cumulative physical effects can further complicate motion‐informed dose prediction.

Even though Geant4 has its advantages, it is not without shortcomings. Calculational efficiency remains its greatest hurdle. Conventional 4D Monte Carlo is computationally expensive because a much finer spatial resolution is required to obtain high statistics. This concern is particularly notable in adaptive workflows or in trying to make a real‐time dose prediction. An illustrative diagram of the Geant4 simulation pipeline, with deep learning for motion‐based dose prediction, is shown in Figure 2. Clinical implementation is limited by factors such as treatment delivery time, adaptive workflow duration, and computational latency. While GPU acceleration and AI‐based surrogate models significantly decrease calculation time, real‐time adaptive carbon‐ion therapy is still in development. These factors are anticipated to gain importance in future FLASH carbon‐ion therapy, where ultra‐high dose‐rate delivery necessitates swift motion monitoring and verification to maintain dosimetric accuracy and the intended FLASH effect.

**FIGURE 2:**
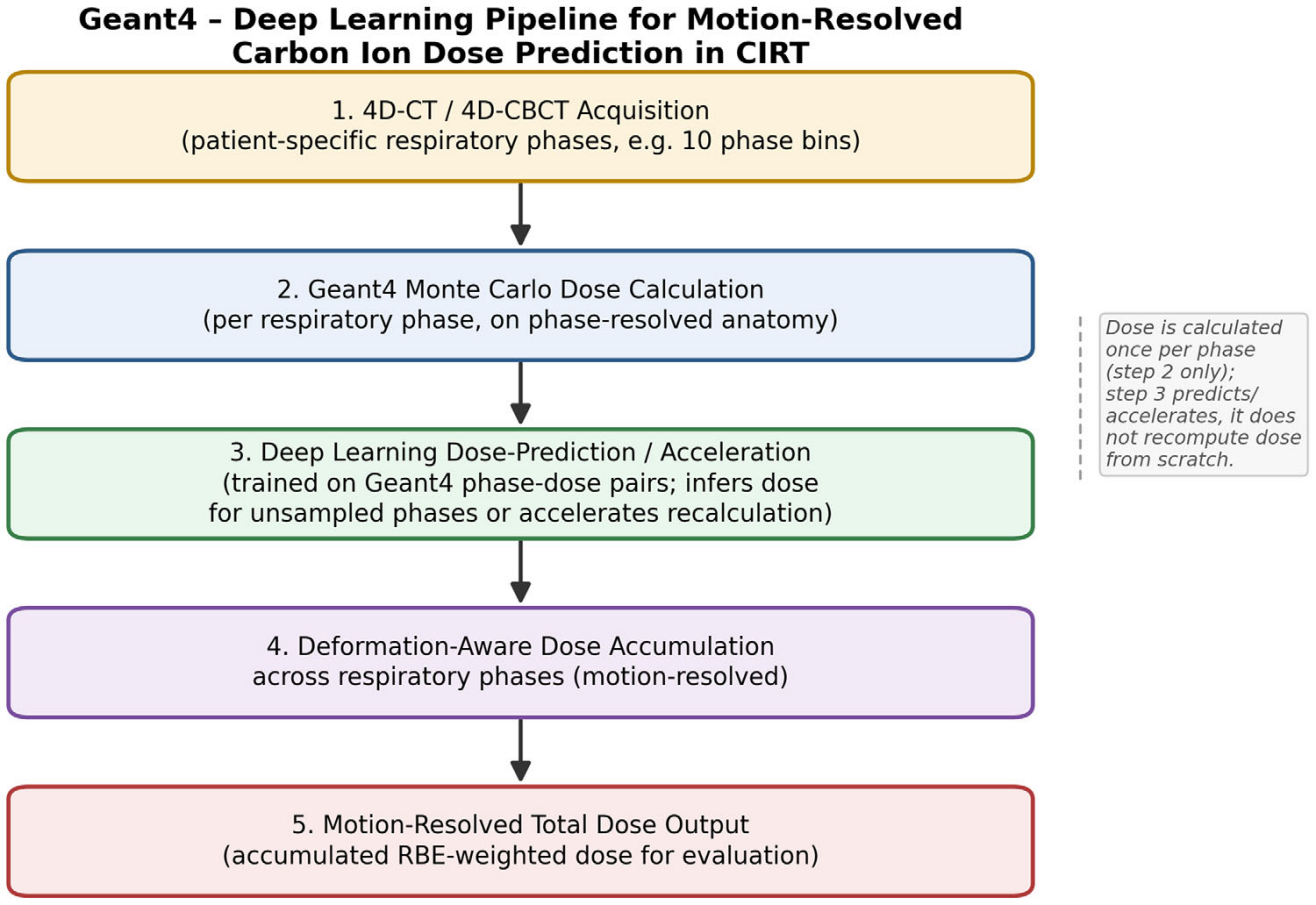
Flowchart showing the integration of Geant4‐based simulation and deep learning models for predicting motion‐affected dose distributions in carbon ion radiotherapy.

In an attempt to overcome this bottleneck, a number of strategies have emerged. Deep learning methods, especially convolutional neural networks (CNNs) and U‐Nets, have been used to generate approximations of the dose distributions by a selected subset of the input simulation parameters using surrogate modeling. With these models, which have been trained on Geant4 with high‐fidelity output, dose can be predicted with high accuracy and quickly across different respiratory cycles.^58^ Deformable image registration (DIR) has similarly been proposed to accumulate dose across respiratory phases by mapping dose calculated on a reference anatomy onto deformed anatomical states. For carbon ions, however, this approach must be applied with caution: because the Bragg peak position is highly sensitive to the density and path length of tissue traversed, dose distributions cannot generally be deformed across anatomical states without recalculation, and full 4D‐MC recalculation on each respiratory phase remains the more reliable approach for accurate dose accumulation in CIRT.

Combining Geant4 with fast analytic dose engines or machine learning (AI)‐aided correction layers has led to the development of hybrid simulation techniques to facilitate performance (near real‐time) without compromising physical accuracy. They consist of precomputed Monte Carlo data that are utilized in training generative AI that can dynamically predict the dose deposition patterns (it should be noted that Sarrut et al.^59^ is a general review of AI applications in Monte Carlo simulation for medical physics, citing a single proton MC application and none specific to carbon ions or Geant4; similarly, Webster et al.^60^ is a general review of computational radiotherapy approaches without a specific AI‐Geant4‐carbon ion connection—both are cited here for the broader methodological context they provide rather than as carbon‐ion‐specific evidence). Other studies investigate cloud and GPU‐accelerated platforms that optimize wall‐time without compromising Monte Carlo precision in four‐dimensional scenarios.^60^ The use of Geant4 within the context of more general simulation pipelines that incorporate artificial intelligence, adaptive feedback, and probabilistic modeling remains a topic of ongoing interest as the field evolves, enhancing the applicability of Geant4 in CIRT. However, further efforts are needed to standardise these hybrid techniques for clinical application and to validate them on various patient anatomies.^59^

## 6 CLINICAL & PRACTICAL IMPLICATIONS

Carbon ion radiotherapy benefits from adaptive replanning under respiratory motion owing to its sharp dose gradients and sensitivity to anatomical changes. Approaches such as respiratory gating and motion modelling have been demonstrated to accurately estimate and correct irregular motions in real time. As an example, Meschini et al^52^ explain how motion predictive models could be used to guide beam gating, and Kubiak delineates the layered rescanning and tumor tracking methodologies applicable in offline adaptive replanning and the developing field of online adaptive radiotherapy during treatment delivery.^8^ Integration of 4D imaging and dose computation can provide a temporally resolved understanding of dose delivery across motion cycles.^61^, ^62^ Techniques employing tools to synchronize beam delivery with 4D CT data sets, for example those mentioned by,^63^ provide an overall picture of dose distribution throughout the phases of motion.^63^ Rietzel and Bert's framework, while foundational, is now 15 years old and was developed primarily for regular, periodic respiratory motion; Steinsberger et al.^64^ provide a modern extension of this motion‐management framework that explicitly addresses irregular respiratory motion, which is more representative of real patient breathing patterns. Such temporal accuracy minimizes the differences between the planned dose and the actual dose. Mori et al^65^ have also compared the gated and ungated planning approaches on pancreatic cancer using 4D CT and indicated significant accuracy improvements with the motion‐managed approach.

Real‐time verification of dose tools has also been reported, such as the one reviewed by Galeone et al., which allows moving targets to be corrected almost in a real‐time scenario^66^. Since the respiratory motion is variable, individual motion profiles necessitate margin adaptation. According to,^67^ integration of the real‐time motion pattern analysis would enable clinicians to plan with customized volumes of target (PTVs), which eliminates the irradiation of healthy tissues unnecessarily. Adaptive radiotherapy frameworks, such as range‐adapted internal target volumes (raITVs), allow PTV tailoring based on motion amplitude and direction.^68^ demonstrated cycle‐based respiratory guidance methods that refined beam delivery timing and margins. Furthermore, Jia et al.^69^ quantified the improvements in organ‐sparing and dose conformity for patients with locally advanced NSCLC using adaptive carbon ion strategies.

## 7 CHALLENGES AND FUTURE DIRECTION

Machine learning algorithms for real‐time, motion‐ and dose‐adaptive applications require large labelled datasets to capture a wide range of respiratory dynamics. Nevertheless, this type of dataset is limited in CIRT. Fossati et al. stress that motion‐rich data should be gathered to enhance the bioinformatic learning used in clinical practice to achieve more adaptation and resilience with the help of AI.^70^ Pakela et al^9^ highlighted the existence of this gap, and they stressed the importance of collaborative data generation to achieve applications of robust machine learning in the field of particle therapy.

RBE‐weighted dose is already incorporated in adaptive planning workflows at leading CIRT centers, including LEM‐ and MKM‐based implementations in RayStation, and recent studies have demonstrated real‐time RBE‐weighted dose computation; the ongoing challenge is therefore not whether RBE can be included in adaptive planning, but how accurately and efficiently spatially variable RBE can be recalculated as anatomy changes during treatment. As explained by,^23^ modern models of RBE are complicated and mostly non‐linear with dynamic conditions. The LET distributions that are variable with respect to motion inevitably require a biologically‐weighted dose modeling to prevent under‐ or over‐treatment of regions. It can be stressed that work by Karger demonstrates that conventional models of RBE can underestimate biological efficacy with different modes of motion.^23^ Research, such as that by,^71^ is attempting to combine NTCP models with biological dose optimisation in the treatment of lung cancer.

Such adaptive strategies as 4D dose re‐computations and optimization of relative biological effectiveness are very computationally demanding, in many cases contradicting the fast schedule of decision‐making within the clinics. As Lebbink et al.^43^ note, there is a challenge in performing real‐time recalculations without adversely affecting clinical throughput. Kim et al.^72^ discussed that reducing the latency in dose recalculation and beam path adaptation is essential to avoid workflow bottlenecks. Fast dose engines and GPU‐accelerated platforms are under development but remain inconsistently implemented across clinics.^73^ To transition from bench to bedside, these innovations require prospective validation and regulatory approval. Motion modelling protocols and multi‐centre trials must be standardised. Chi et al.^45^ highlight that the lack of rigorous clinical evidence will lead to a protracted adoption of even promising technologies, as safety and reproducibility will be at issue. Future experiments, such as those cited by Jia et al.^69^ are required to confirm the safety and effectiveness of adaptive strategies. Regulatory pathways should also adapt to the complexities of biologically and motion‐guided adaptive treatment approaches, as highlighted by Pakela et al.^9^. These uncertainties have a direct impact on the routine clinical implementation of radiotherapy. Specifically, uncertainty in Relative Biological Effectiveness (RBE) modelling may affect the selection of prescription doses. Additionally, the limited availability of motion‐rich datasets constrains the generalisability of artificial intelligence models. Computational latency continues to pose a challenge to online adaptive radiotherapy, thereby limiting its widespread clinical application. Most integrated Geant4–deep learning adaptive carbon‐ion radiotherapy frameworks are currently at Technology Readiness Levels (TRLs) 3–5, which are primarily based on simulation or preclinical validation. Routine clinical implementation requires multicentre validation, integration into clinical workflows, regulatory approval, and prospective clinical trials.

## 8 CONCLUSION

The studies reviewed here are, almost without exception, simulation‐based, retrospective, or preclinical, and most integrated Geant4–deep learning frameworks for motion‐adaptive carbon‐ion radiotherapy remain at Technology Readiness Levels (TRL) 3–5. Within that limit, the trajectory of the field is consistent: Geant4‐based Monte Carlo simulation provides high‐fidelity modelling of particle transport and dose deposition under respiratory motion, and deep learning approaches—particularly when constrained by physical priors, as in physics‐informed neural networks—have repeatedly been shown to approximate these Monte Carlo dose distributions with substantially reduced computation time. He et al.^18^, for example, demonstrated near‐real‐time dose prediction with high agreement to Monte Carlo reference doses, though on a limited, retrospective patient cohort, and purpose‐built architectures such as DOTA and C‐DOTA point toward further refinement of this approach rather than a solved problem. What is not yet established is direct, prospective clinical validation of these hybrid frameworks specifically under 4D respiratory motion in CIRT: published evidence to date supports feasibility and computational promise, not clinical readiness. Realising the potential of this combination—motion‐compensated, biologically weighted treatment planning delivered at clinically usable speed—will require standardised benchmarking datasets, multicentre prospective validation, integration into existing adaptive workflows, and regulatory engagement, none of which has yet been demonstrated at scale. Until these steps are taken, the appropriate framing for this body of work is one of a promising but early‐stage research direction rather than a near‐term clinical solution.

## AUTHOR CONTRIBUTIONS

**Chai Hong Yeong**: Conceptualization; methodology; investigation; writing—original draft. **Peng Li**: data curation; investigation; writing—review and editing. **Jianhua Ding**: formal analysis; writing—review and editing.

## CONFLICT OF INTEREST STATEMENT

The authors declare no conflict of interest.
